# Coupling movement with imagery as a new perspective for motor imagery practice

**DOI:** 10.1186/1744-9081-9-8

**Published:** 2013-02-20

**Authors:** Aymeric Guillot, Kevin Moschberger, Christian Collet

**Affiliations:** 1 Centre de Recherche et d’Innovation sur le Sport, EA 647, Université de Lyon, Université Claude Bernard, Lyon 1, Performance Motrice, Mentale et du Matériel (P3M), 27–29 Boulevard du 11 Novembre 1918, Villeurbanne, Cedex, 69622, France; 2 Institut Universitaire de France, 103 Boulevard Saint-Michel, Paris, 75005, France

**Keywords:** Movement imagery, Dynamic imagery, Motor cognition, Motor performance, Mental imagery

## Abstract

**Background:**

Recent data support the beneficial role of gesturing during mental practice. The present study examined whether coupling motor imagery (MI) with some movement sequences (dynamic imagery condition) impacted motor performance to a greater extent than performing MI while remaining motionless.

**Methods:**

A group of active high jumpers imagined their jump both with and without associated arm movement. Three outcome variables were measured: the number of successful attempts, the temporal congruence between MI and actual jump performance, and the technical quality of the jump.

**Results:**

Data revealed that dynamic imagery enhanced both MI quality and temporal congruence between MI and motor performance, and further improved the technical efficacy of the jump. Athletes also reported more vivid representation while coupling MI with actual movement.

**Conclusions:**

These data support the hypothesis that performing dynamic imagery might contribute to enhance MI quality and efficacy, and sketch potentially fruitful new directions for MI practice.

## Background

Motor imagery (MI) is one of the remarkable capacities of the mind enabling everyone to mentally simulate an action without engaging in actual physical execution [1]. MI and physical practice share similar neural substrate, albeit corresponding neural networks are not totally overlapping [2-5], hence supporting the principle of functional equivalence [6]. Based on this concept, the efficacy of an imagery intervention is thought to depend on this abstract idea of neural similarity between MI and motor performance. From a practical viewpoint, previous experimental research provided evidence that MI contributes to improve performance in athletes and to promote recovery from injury (for reviews, [7-12]). Interestingly, unless MI is congruent with physical practice, it will not be as effective in achieving its desired effects. For example, it is now well-known that physical experience is important before engaging in MI. At a neural level, it may probably contribute to a greater overlap between MI and motor performance. So far, several MI models have been designed to provide a detailed description of the key-components of the MI content to ascertain its efficacy, and contribute to develop more effective imagery interventions (e.g., the PETTLEP model [6] and the MIIMS model [9]).

Among others, motor execution issues during MI should be close to those related to actual execution, that is, the spatial and temporal characteristics should be preserved during MI [13]. For instance, preserving the temporal accuracy during mental rehearsal is required to avoid harmful alterations of the actual movement timing. Indeed, imagining a motor sequence either at a slower or faster pace might affect the corresponding actual movement speed [14-16]. Conversely, MI speed is not a crucial factor when integrating in imagery sequences with a motivational outcome, and some athletes occasionally reported using voluntarily slow, real-time and fast MI to achieve different outcomes [17]. Likewise, athletes frequently imagine their pending motor performance while adopting the same position as that required by its physical execution, which is believed to facilitate the MI process [18,19]. The environment or context in which MI is performed is also likely to facilitate the mental operations required to form accurate and vivid mental images [20,21].

With reference to the definition we gave in the above paragraphs, the participants are usually requested to perform MI in the absence of movement during mental rehearsal sequences [22]. A large amount of imagery interventions therefore decoupled MI from the action, and experimental designs even checked with electromyography that participants did not contract any muscles during MI. A subliminal muscle activity has been, however, frequently recorded during MI, which suggests that the motor command is probably not fully inhibited. Guillot et al. [23] and Lebon et al. [24] reported that the subliminal muscle responses during MI of concentric, isometric and eccentric contractions typically mirrored the configuration of the muscle activity recorded during actual practice. This issue contributes to support that mental representation is very close to actual practice, and that moving while imagining a given action might be possible. Moreover, the recent “motor cognition” theory considers MI as being placed along a continuum where intentional movement is on one side and representation at the opposite [25]. Accordingly, MacIntyre and Moran [26] claimed that MI is coupled with motor execution, and thus that movement is possible *during* imagined actions. Practically, enhancing imagery effectiveness by incorporating movement during MI has been promoted early on in applied studies [27,28], and further discussed in theoretical models of imagery [6]. For instance, Holmes and Collins [6] stated that athletes should be actively involved in the imagery experience, for example by involving sporting implements and making movements as necessary. Despite this previous study, however, few experiments examined the association of MI with movement, whether it is moving while imagining or imagining while moving. Vergeer and Roberts [29] investigated the effectiveness of performing MI during stretching, by asking participants imagining leg movements during stretching, i.e. continuously flexing the knee and bringing the heels toward the buttocks. They found a positive correlation between improved flexibility and vividness ratings, hence suggesting that coupling MI with stretching might have contributed to extend the range of motion or increase the duration of the stretch. In a seminal paper, Callow et al. [21] later provided evidence that high level junior skiers who moved their body from side to side during MI (dynamic imagery group), as if they were actually racing, experienced the most vivid imagery and increased their confidence to perform the task. Other authors also lend support for the dynamic aspect of imagery, and showed that moving during imagery might result in greater improvements in performance compared to remaining motionless [30]. A more recent study confirmed the beneficial role of gesturing during spatial problem solving, although MI should not be confounded with spatial problem solving [31]. Specifically, the authors provided evidence that performing spontaneous hand movements during mental rotation improved performance. The authors argued that the production of similar co-thought gestures could facilitate other types of concurrent mental tasks - such as MI - as well. Finally, as underlined by Lorey et al. [32], we are all familiar with pictures of athletes moving while imagining their subsequent performance during pre-performance routines. While athletes claim that moving may prime and facilitate MI, whether coupling MI and actual movement contributes to both improving subsequent motor performance and achieving temporal congruence between MI and motor performance has not yet been experimentally addressed.

The present study was designed to determine whether coupling MI with actual movement (dynamic imagery) impacts motor performance to a greater extent than performing MI without any overt body movement, and further contributes to achieve the temporal congruence between MI and motor performance. Based on the assumption that MI and overt movement are intimately related one to another both at the behavioral [25,26] and neurophysiological level [33], we postulated that coupling MI with actual movement might lead to improvements in technical aspects compared to performing MI while remaining motionless and further result in better temporal congruence between MI and actual times.

## Methods

We used a within-subjects design allowing reduction in error variance associated with individual differences. However, to avoid that participation in one imagery condition affects performance in the other imagery condition, all MI trials were scheduled in a random order.

### Participants

A sample of high jumpers was recruited for the experiment as this motor skill is appropriate for imagery training [34], and elite high jumpers frequently move while imagining their motor performance during pre-performance routines. A total of 12 right-handed healthy high jumpers aged between 16 and 25 years old (mean age: 20.42 ± 3.61 years, 6 women) took part in the study, which was approved by the Research Ethics Committee of the University. All athletes were competing at national level events since 5 to 14 years, and with personal bests ranging from 156 cm to 218 cm. They were free of any recent injury affecting motor skills, balance, and had normal or corrected-to-normal vision. The procedure of the experiment was explained to the participants, but no information was provided about the objectives of the study or the dependent variables of interest.

### Procedure and motor task

A first session was scheduled one week before the experiment to select the athletes and determine their knowledge about MI. In particular, they were questioned about the frequency and the nature of their imagery use, in order to exclude athletes who regularly performed imagery routines including movements, and/or who would have been unfamiliar with motionless imagery. Participants were also given descriptions of internal visual imagery, external visual imagery, and kinaesthetic imagery, and were advised that they will be able using kinaesthetic imagery in combination with whichever visual support they found beneficial. All athletes self-reported using more frequently and easily internal visual imagery along with kinaesthetic imagery. This combination was therefore considered in the imagery scripts (see below).

The individual imagery ability of the participants was evaluated to ensure that the sample did not include athletes with extremely high or low imagery ability, which could have influenced the capacity to imagine in real time. The revised version of the Movement Imagery Questionnaire (MIQ-R, [35]) was completed to measure the individual ability to form kinaesthetic and visual mental images. The MIQ-R consists of an 8-item self-report questionnaire, in which the participants rate the vividness of their mental representation using two 7-point scales. We measured the individual ability to form visual images using the first series of items (from 1 = “*very hard to see*” to 7 = “*very easy to see*”), the ability to perceive the sensations usually elicited by the movement during kinaesthetic imagery with the second (from 1 = “*very hard to feel*” to 7 = “*very easy to feel*”). The MIQ-R has demonstrated adequate reliability and validity with alpha coefficients of .79 for both subscales.

**Figure 1:**
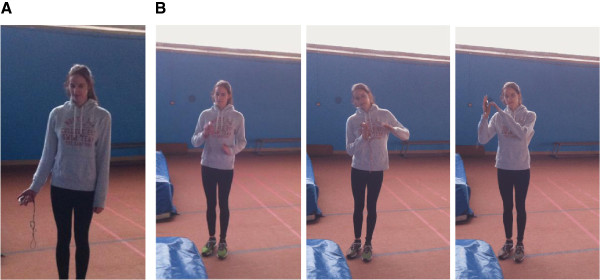
Motionless (A) and dynamic (B) imagery conditions.

In accordance with the coaches, athletes were asked to imagine and actually jump over a bar placed at 90% of their personal best, which had been performed during the season. After four warm-up jumps, each athlete randomly performed a total of 10 actual jumps and 10 MI trials within a single practice session. Practically, the first trial was always a physical practice trial, in order to avoid participants imagining doing a task without having performed it physically beforehand. Half of the MI trials were performed while remaining motionless (*motionless imagery*), the other five trials being coupled with actual movement (*dynamic imagery*), i.e. athletes were asked to freely mimic the actual jump phases with their arms. All participants performed MI in a position which was compatible with the motor skill, i.e. in a standing position. Practically, we define dynamic imagery as a specific sequence of MI which is accompanied by external movements miming in part those which are mentally represented, in particular specific movement features related to temporal or spatial invariance. Athletes were thus asked to mimic the actual movement using simple upper-limb movements, but without engaging in the actual motor act and while keeping the lower limbs motionless. The main functional aim of associating movement to MI was to provide sensory feedback including somatosensory inputs likely to facilitate the mental representation of the jump, and further help participants to formalize the spatio-temporal configuration of the movement. Specifically, movements performed during MI brought temporal boundaries, and thus had a temporal function to facilitate calibration of time. Finally, lower-limb movements were excluded to limit the intensity of the motor act during the mental representation (Figure 1). More generally, athletes were asked to consider the body as a generator of forces, and therefore to combine internal visual imagery with kinaesthetic imagery. A detailed imagery script based on previously published imagery research was read to ensure that the participants followed similar instructions throughout MI sessions^a^. To collect MI times, participants hold a timer in their non-dominant hand to ensure that chronometric measures reflected only the mental representation of the movement, and did not include other static images such as the preparation phase. Before the experiment, we checked that participants did not feel uncomfortable with this procedure.

### Measures

Three outcome measures were used as dependent variables to evaluate the efficacy of MI. The temporal congruence between MI and physical performance was first recorded as a reliable marker of MI accuracy [36-38]. Actual and MI times were thus recorded to test the individual ability to imagine in real time. Actual times were recorded by the same experimenter, whereas MI times were recorded by the athletes themselves, who triggered a timer as early as they imaged the first move, and stopped the timer at the end of the jump, when falling down on the carpet. They never received any feedback on MI times during the session to prevent any influence on subsequent trials. Secondly, the number of successful jumps was considered for the motionless imagery condition vs. the dynamic imagery condition. Thirdly, motor performance was evaluated by considering the number of successful trials and by providing efficiency ratings of technical motor skill components that are known to influence jumping height. This latter evaluation was made by two expert trainers known for their expertise in high jump. These measures were adequate as MI has been shown to impact technique without necessarily resulting in immediate higher jump height [34]. To do so, athletes were filmed by a digital video camera (SANYO VPC-HD2000A, 30 Hz). Then, the two experts, who were not aware of the detailed experimental conditions, and did not have a chance to see the athlete during MI, rated the quality of the jump. To match live conditions, they independently watched each jump only once, and then rated each trial using a 0 (poorest performance) to 10 (best performance) scale. For each of the four selected performance items, marks of both experts were averaged to provide a global score. Experts first rated the quality of the run *approach* before the curve, related to the amount of speed and the number and stride frequency. The second item was the quality of the *curve*, including four to six strides. The quality of the *impulsion* was rated with reference to the take-off angle, body segments alignment and knee position of the free limb. Finally, *bar clearance* was rated by evaluating the effectiveness of the movements of shoulders, knees and feet. An overall technique score was also considered by averaging the four subscores provided by the two experts.

Finally, individual debriefings were scheduled to control that MI instructions were respected, and to determine whether participants encountered difficulty in forming mental images. Accordingly, participants were required to rate the vividness of the mental images they attempted to form during each condition, using a Likert-type scale (from 1 = “*unclear and inaccurate mental representation*” to 6 = “*perfectly clear and vivid mental representation*”). They were also asked to report the experimental condition in which they formed more easily mental images of the movement.

### Data analysis

There was no significant gender difference on MI and actual times or on any rating provided by the two experts, and thus there was no need to subdivide the group by gender. One-way repeated measures analyses of variance (ANOVA) with two conditions (motionless imagery vs. corresponding actual times, and dynamic imagery vs. corresponding actual times, respectively) were performed to compare chronometric data. Repeated measures ANOVAs were also computed to compare the number of successful jumps (“hit/miss”), the overall technique score, and expert ratings in each experimental condition. Data were then split into hits and misses and an additional 2 (Dynamic vs. Motionless imagery) x 2 (Hit vs. Miss) repeated measure ANOVA was performed to compare actual and MI times. Effect sizes, including partial eta-squared (η^2^) for ANOVA results are also provided. The results are presented as a mean (standard deviation), and a level of *p* < 0.05 was considered critical for assigning statistical significance.

## Results

### Individual imagery ability

Mean MIQ-R scores (SD) were 39.50 (3.90). Mean score was 21.58 (1.98) for the visual subscale, and 17.92 (2.78) for the kinaesthetic subscale. Visual scores were higher than kinaesthetic scores (*F*(1,11) = 20.02, *p* < 0.001, η^2^ = .64). There was no participant with extremely high or low MIQ-R score (two SD above or below the mean), and current MIQ-R mean scores were comparable to those observed in previous MI studies [15,23].

### Motor imagery and actual times

**Figure 2:**
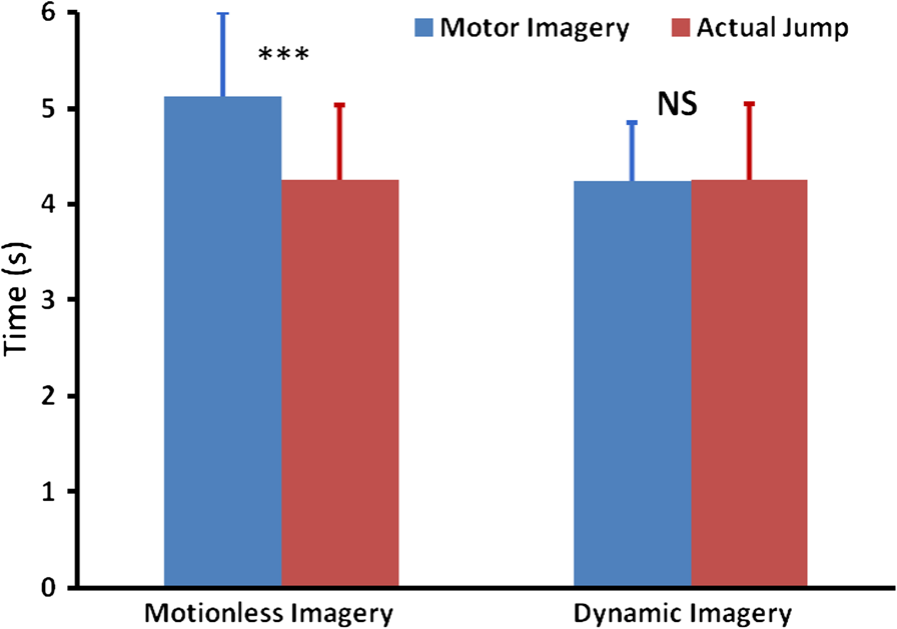
**Mean (SD) Actual and motor imagery times.** A significant difference was found between imagery and actual times when participants performed motionless imagery. In contrast, they achieved temporal congruence during dynamic imagery. ***: p < 0.001; NS: non-significant.

When MI was performed while remaining motionless, mean MI time was 5.12 s (0.88), and mean corresponding actual time was 4.24 s (0.78). The ANOVA revealed that this difference reached significance (*F*(1,11) = 24.72, *p* < 0.001, η^2^ = 0.69, Figure 2). When MI was coupled with actual movement, mean MI time was 4.26 s (0.79), and mean corresponding actual time was 4.25 s (0.62). The ANOVA did not reveal any difference between these times (*F*(1,11) = 0.02, *p* = 0.91, η^2^ = 0.001, Figure 2).

An additional 2 (Dynamic vs. Motionless imagery) x 2 (Hit vs. Miss) repeated measure ANOVA was performed to compare actual and MI times after splitting jump performance into hits and misses. Data revealed a main effect of the imagery condition (Dynamic vs. Motionless imagery, *F*(1,116) = 8.98, p = 0.003), but no effect of performance (hit vs. miss, *F*(1,116) = 0.97, p = 0.33) nor significant imagery condition x performance interaction (*F*(1,116) = 0.10, p = 0.75).

### Outcome measures

**Figure 3:**
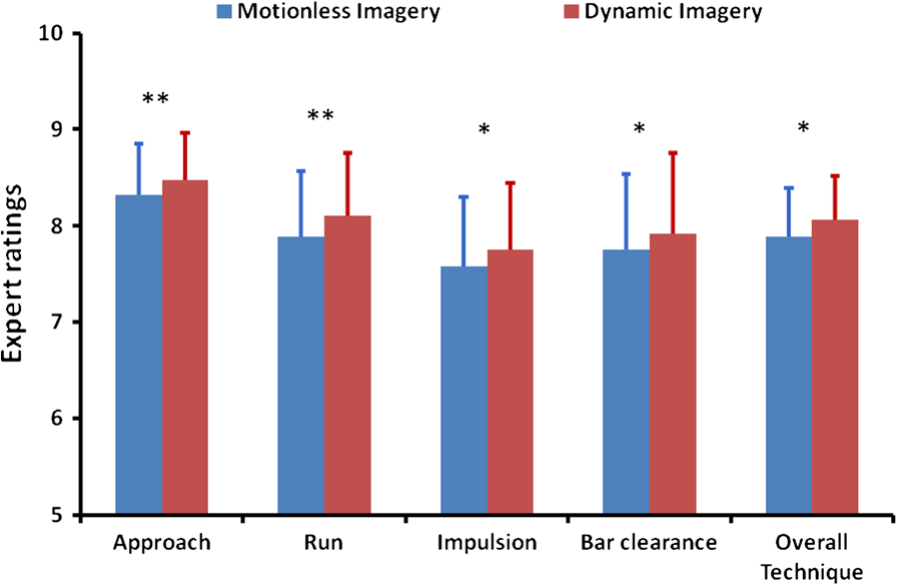
**Mean (SD) expert ratings during motionless and dynamic imagery.** Greater performance was systematically obtained during dynamic imagery than during motionless imagery. *: p < 0.05; **: p < 0.01.

Evaluating motor performance in terms of hit and miss revealed a higher percentage of success during dynamic imagery (45%) than during motionless imagery (35%). Comparing expert ratings measuring the quality of the jump then revealed systematic greater performance during dynamic imagery compared to motionless imagery (Figure 3). Mean overall technique scores were 7.89 (0.51) during motionless imagery and 8.06 (0.46) during dynamic imagery, the difference reaching the statistical threshold (*F*(1,11) = 4.97, *p* < 0.05, η^2^ = 0.31). Mean ratings for the *approach* item were 8.48 from 10 (0.49) during dynamic imagery and 8.33 (0.53) during motionless imagery (*F*(1,11) = 9.58, *p* < 0.005, η^2^ = 0.47). Likewise, greater scores were obtained during dynamic imagery than during motionless imagery for the *curve* item (*F*(1,11) = 9.73, *p* < 0.005, η^2^ = 0.47), mean scores being 8.11 (0.65) and 7.89 (0.68), respectively. Greater scores were also found during dynamic imagery than during motionless imagery for the *impulsion* item (*F*(1,11) = 3.91, *p* = 0.04, η^2^ = 0.26), mean scores being 7.75 (0.70) and 7.58 (0.72), respectively. Finally, greater scores were obtained during dynamic imagery than during motionless imagery for the *bar clearance* item (*F*(1,11) = 3.50, *p* < 0.05, η^2^ = 0.24), respective mean scores being 7.93 (0.83) and 7.76 (0.78).

### Assessment of imagery use

During the debriefings following MI sessions, all participants reported using motor imagery as outlined in the scripts. All participants combined internal visual, auditory, and kinaesthetic imagery without switching to external visual imagery, nor changing the imagery script to suit individual needs. Accordingly, they were able to report the series of movements with an explicit knowledge of each key-component of the physical execution. Finally, respective mean ratings given by athletes when evaluating the vividness of MI were 4.67 (0.98) during dynamic imagery and 3.58 (1.24) during motionless imagery. The ANOVA revealed that the difference reached significance (*F*(1,11) = 14.19, *p* < 0.003, η^2^ = 0.56).

## Discussion

The present study was designed to test whether integrating actual movement during MI was likely to reinforce its well-known beneficial effects, and whether it may contribute to achieve the temporal congruence. The main results revealed that moving while imagining enhanced the quality and the whole timing of MI and, more particularly, the temporal exactness of the approach run before the take-off of the jumping. The technical efficacy of the jump was also improved, along with an increased number of successful trials, and athletes reported forming mental images of the movement more easily and accurately during the dynamic imagery condition. Altogether, these data support that coupling MI with actual movement might contribute to reinforce MI quality and, therefore, its expected efficacy.

Among the important prerequisites in developing MI training programs, there is now ample evidence that athletes must achieve a temporal congruence between MI and physical practice when they use MI to perform/rehearse movements [16,37] for reviews]. The difficulty to preserve the temporal features of the movement during MI has also been taken as imagery impairment [38,39], and recording MI time is a reliable technique for assessing MI ability [40]. Interestingly, present findings provided evidence that coupling MI with actual movement-related gestures, i.e. performing dynamic imagery resulted in a better temporal congruence between MI and physical practice than performing MI while remaining motionless, hence suggesting that MI was temporally more accurate. Dynamic imagery provides time boundaries that would probably make the preparation phase before execution more effective. This temporal accuracy came probably from subdividing the whole movement into several sub-sequences associated with time boundaries, which are mentally reproduced, thus giving temporal references to athletes. Achieving such temporal congruence is necessary as changes in imagery speed can rapidly affect the subsequent actual speed [14], even for highly automated motor tasks where the duration is very much set and controlled [15,16]. During high jump, athletes must visualize run-up without any feedback regarding the optimal horizontal velocity and adjustment of the length, height, and speed of their strides to prepare the take-off. Interestingly, present data suggest that dynamic imagery helped them to calibrate the jump, and more particularly the run-up, through a greater internal representation of its rhythm and tempo. The improved temporal accuracy could also result in increased imagery vividness and therefore a richer representation displayed in participants’ working memory [21]. Practically, the spatio-temporal features of the task are likely to be more closely reproduced when athletes mimic their performance during MI, which might theoretically contribute to improve subsequent actual jumps. Experimental validation of this working hypothesis will certainly be the next step of dynamic imagery research.

As suggested by Olsson et al. [34], motor performance was not only measured in terms of successful or missed jump but also using critical technical components that influence jumping height. Data first showed that the rate of successful jumps tended to be better when coupling MI with actual movement than when performing motionless MI. The comparison of expert ratings measuring the technical quality of the jump confirmed the benefits provided by the dynamic imagery condition. The most significant differences were observed during the approach and the curve, hence supporting that moving while imagining contributes to better calibrate the run-up. Hence, present findings confirmed that MI is beneficial for skills that require complex movements such as bar clearance [34,41], but also supported its efficacy for enhancing the internalization and calibration of the approach, which is also a critical factor of performance, albeit less complex in terms of technique.

Previous data showed that MI is a cost-effective and valuable complement, but not a substitute, to physical training, and that combining MI and subsequent physical practice is more efficient than physical practice or MI alone [7,9,42]. Interestingly, looking at the effect of MI on motor recovery even proposed that the benefits of MI are essentially due to combined physical and mental practice, while MI alone does not necessarily result in greater performance [43]. In accordance with these assumptions, and based on present data as well as suggestions from previous researchers [7,21,26,27,29-31], we state that using dynamic imagery, i.e. performing simple upper-limb movements which integrate mainly temporal features of the actual practice during MI, is a potentially fruitful direction to perform MI with important advantages. First, moving provides actual feedback, thus offering an effective solution to the main limitation of MI, that is, the absence of proprioceptive feedback. Accordingly, we postulate that even if athletes only mimic their actual performance with their arms, such limited feedback is likely to improve the individual ability to perform kinaesthetic imagery. This hypothesis was somewhat confirmed by athletes’ self-estimation of their MI vividness. They significantly reported forming more accurate mental images of the movement while moving than while remaining motionless. Second, it might contribute to improve the ability to achieve temporal congruence between MI and actual performance. In the case of high jump, this is particularly effective for the run-up. Third, coupling MI with actual practice is in accordance with the motor cognition approach supporting that there is a continuum between motor preparation/execution and MI.

Interestingly, we observed that performing dynamic imagery is beneficial in experienced athletes, while one might have expected a more limited effect due to such level of expertise. We believe that moving while imagining is likely to enhance the mental representation and the calibration of the run-up, which usually remains a difficult task even in confirmed athletes. Indeed, athletes must resolve a complex relationship between the speed of the approach running and the vertical velocity to be produced for jumping. Moving while imagining might therefore contribute to stabilize a given tempo for this part of the whole movement. Practically, this result suggests that dynamic imagery might be used regardless of the level of expertise.

For a more theoretical viewpoint, we postulate that moving while imagining may have emphasized the degree of behavioral matching, and possibly the functional equivalence between MI and motor performance, which may contribute to explain the positive effects of dynamic imagery. As suggested by the PETTLEP model of MI, interventions should simulate, as closely as possible, all aspects of participants’ execution situations [44]. Allowing athletes to perform dynamic imagery, at least in some occasions, might thus help them to achieve this goal. Obviously, considering that MI and action can occur simultaneously also raises some theoretical concerns. First, we state that athletes could move while they imagine their motor performance, which is conceptually different from imagining while they are moving. In the first case, limited motor execution would merely supplement the MI input [26], while in the second, MI might occur as an epiphenomenon during the motor execution process. Second, one may question whether dynamic imagery is a kind of imagery practice *per se*, strictly speaking, as it challenges the common belief that MI occurs in the absence of sensory input. Nikulin et al. [45] introduced the concept of quasi-movements, defined as volitional movements which are minimized by the participant to such an extent that finally they become undetectable by objective measures. Interestingly, they hypothesized that the procedure of learning how to perform quasi-movements (by the successive reduction of movement strength to a complete muscular quiescence) might represent a transition process between motor execution and MI. As outlined by MacIntyre and Moran [26], we state that in every case, performing dynamic imagery requires to reconsider our definitions of MI to encompass movements that can occur during mental practice.

Spurred by these findings, determining the efficacy of dynamic imagery in larger samples and during competition settings is warranted. Some limitations are associated with this study. First, the sample size was rather small with 12 athletes. A second, and potentially more serious limitation, is that only five trials were performed in each imagery condition, thus precluding from general conclusions in terms of performance enhancement. Despite this, the improvement of the technical execution of the motor skill promotes the use of dynamic imagery, and future studies should test this working hypothesis experimentally. Unexpectedly, athletes passed the bar in few attempts, although we enrolled qualified jumpers. We might explain this result by the fact that they were asked to jump over a bar placed at 90% of their personal best, which had been performed lately during the previous competition period. As the experiment was scheduled early in the new competition period, we assume they still did not achieve their optimal level of performance, and therefore the level of difficulty was probably set slightly over 90% of their personal best previous performance. As another limitation of our study, we must acknowledge that we reported the effectiveness of dynamic imagery of a high-technical sporting skill. Although this finding is of importance, we must also consider more simple movements which could be easily learnt in less time and performed by healthy participants who are not necessarily sport athletes. Finally, we did not look at whether MI might contribute to improve motor performance after a training period, and did not include a control condition which is usually essential before drawing general conclusions. Due to the characteristics of the sample (level of expertise, number of athletes), as well as time lack which prevented us from spanning the study over a longer period during the new competition period, we were not able including a control condition. Including a condition with performance of actual movement without any MI may thus be particularly helpful in future studies.

## Conclusions

The present within-subjects design showed that performing dynamic imagery might be of interest in a sample of athletes. Firstly, integrating some movements during MI may contribute to improve the ability to achieve temporal congruence between MI and actual performance, which has been shown to positively influence the efficacy of MI. Secondly, data tend to suggest that the technical efficacy of the jump also improved, along with an increased number of successful trials, therefore opening a way for fruitful imagery applications in such populations of athletes.

## Consent

Written informed consent was obtained from the participant for publication of this report and any accompanying images.

## Endnotes

^a^A copy of the imagery script is available from the corresponding author upon request.

## Abbreviations

ANOVA: Analysis of variance; MI: Motor imagery; MIIMS: Motor imagery integrated model in sport; MIQ-R: Movement Imagery Questionnaire – Revised version; PETTLEP: Physical, Environment, Task, Timing, Learning, Emotion and Perspective.

## Competing interests

The authors declare that they have no competing interests.

## Authors’ contributions

AG conception and design, data analysis and interpretation, writing of the manuscript, final approval of the submitted version. KM Conception and design, data acquisition and collection. CC Conception and design, data analysis and interpretation, revision of the manuscript. All authors read and approved the final manuscript.
